# Supplementary material for: Oxygen minimum zone cryptic sulfur cycling sustained by offshore transport of key sulfur oxidizing bacteria
Source: Nat Commun. 2018 Apr 30;9:1729. doi: 10.1038/s41467-018-04041-x (PMC5928099; doi:10.1038/s41467-018-04041-x)
Supplement: Supplementary file 1 — Supplementary Information [file 41467_2018_4041_MOESM1_ESM.pdf]

SUPPLEMENTARY INFORMATION

for

Oxygen minimum zone ‘cryptic sulfur cycling’ sustained by offshore transport of key sulfur oxidizing bacteria

Callbeck et al.

Contents

File contains a discussion on the specificity of the new GSO131 probe, eight figures and four tables, including captions and references.

Supplementary Discussion

Specificity of the GSO131 probe

The probe GSO131 was designed and tested on the latest available 16S rRNA dataset provided by the SILVA project (see Methods for details). Supplementary Table 3 indicates the specificity and coverage of the GSO131 CARD-FISH probe. The GSO131 probe was designed to be specific for the dominant SUP05 ecotype at station U1 (see also Fig 3). The GSO131 probe that was used in combination with the two additional competitor oligonucleotide sequences excluded outgroup hits, such as those affiliated to the sister lineage Arctic96BD-19. To ensure the specificity of the probe we performed a double hybridization using the SUP05 GSO131 probe, in addition to a probe designed to separately distinguish the Arctic96BD-19 clade (called GSO1290, which was designed in silico to target full-length 16S rRNA gene sequences recovered at the non-sulfidic station 378, Supplementary Table 1). The double CARD-FISH result, shown in Supplementary Figure 8, shows no detectable overlap between the two probes as indicated by the two distinct hybridization signals. We are therefore confident that the specificity of the GSO131 probe is high and that no cross-hybridization occurs outside of the SUP05 clade based on the sequences highlighted in Figure 3. The multiple probe hits stem from very closely related SUP05, most likely from different strains of the same species.

The same probe and competitor oligonucleotides applied at station U1 were used at offshore stations, thus reducing or eliminating the chances of Arctic96BD-19 and other cross-hybridization mismatches (See probe match for GSO131 Supplementary Table 3).

Moreover, to test for the presence of an indigenous offshore population of SUP05 bacteria (i.e. a population distinct than the one identified in coastal waters) we performed a separate 16S rRNA gene analysis at an offshore station, station 378. Station 378, located further south of the eddy (coordinates -13.75 °N -76.64 °E; Table S1), was non-sulfidic and had similar temperature-salinity characteristics as station L2. Hence station 378 was not influenced by cross-shelf transport at the time of sampling. We attempted to recover SUP05 16S rRNA genes from waters within the OMZ at station 378 (83 m depth), however, were unable to amplify and recover SUP05 affiliated genes in our clone library preparations using the universal bacterial 16S rRNA gene primer set GM3f/GM4r. To improve the detection limit and recoverability of full-length GSO sequences, we designed a more specific GSO forward primer GSO1f (designed in silico to target SUP05 and Arctic96BD-19 clades in the SILVA database) and used this in combination with the universal bacterial reverse primer GM4r. However, only sequences affiliated to Arctic96BD-19 bacteria were recovered, no sequences affiliated to SUP05 were found (Arctic96BD-19 sequences were added to the phylogenetic tree in Figure 3(St. 378 Sequences). The lack of recoverable SUP05 sequences (using two independent primer sets) reveals no evidence of an indigenous or distinct offshore SUP05 population.

Our results demonstrate that the GSO131 probe covers the SUP05 diversity in the Peru Upwelling, while excluding outgroup hits with Arctic96BD-19 bacteria. Moreover, our findings highlight that the Peru Upwelling, including the offshore OMZ is dominated by a single SUP05 species-level clade, ^{U}Thioglobus perditus, originating from sulfidic coastal waters.

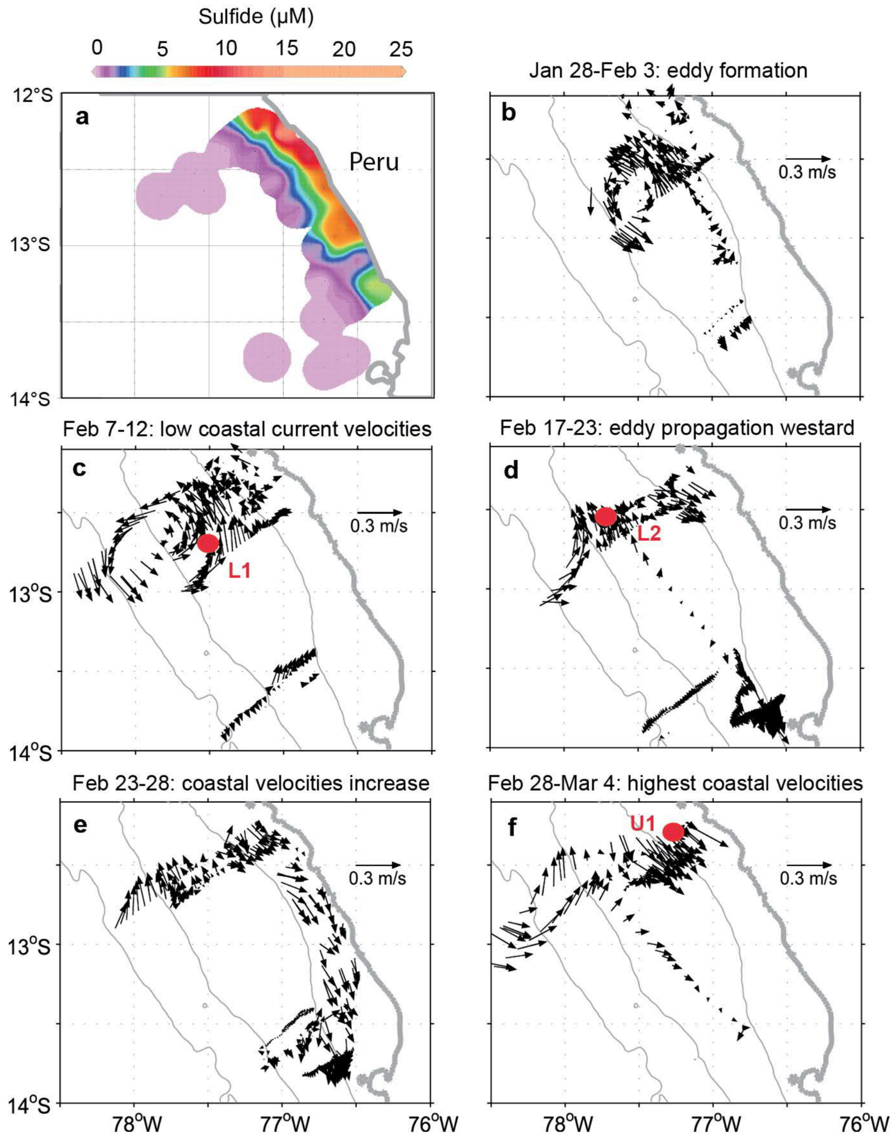

Supplementary Figure 1. Development and propagation of a subsurface mesoscale eddy: (a) Highest bottom water sulfide concentration in the 26.1 and 26.2 kg m^{-3} range from February-March, 2013. (b-f) Snapshots of the subsurface current velocities during the formation and propagation of a lower shelf forming mesoscale eddy. The red circles indicate the main stations sampled within the given time period. Full details related to the eddy hydrodynamics are presented in Thomsen et al., [1].

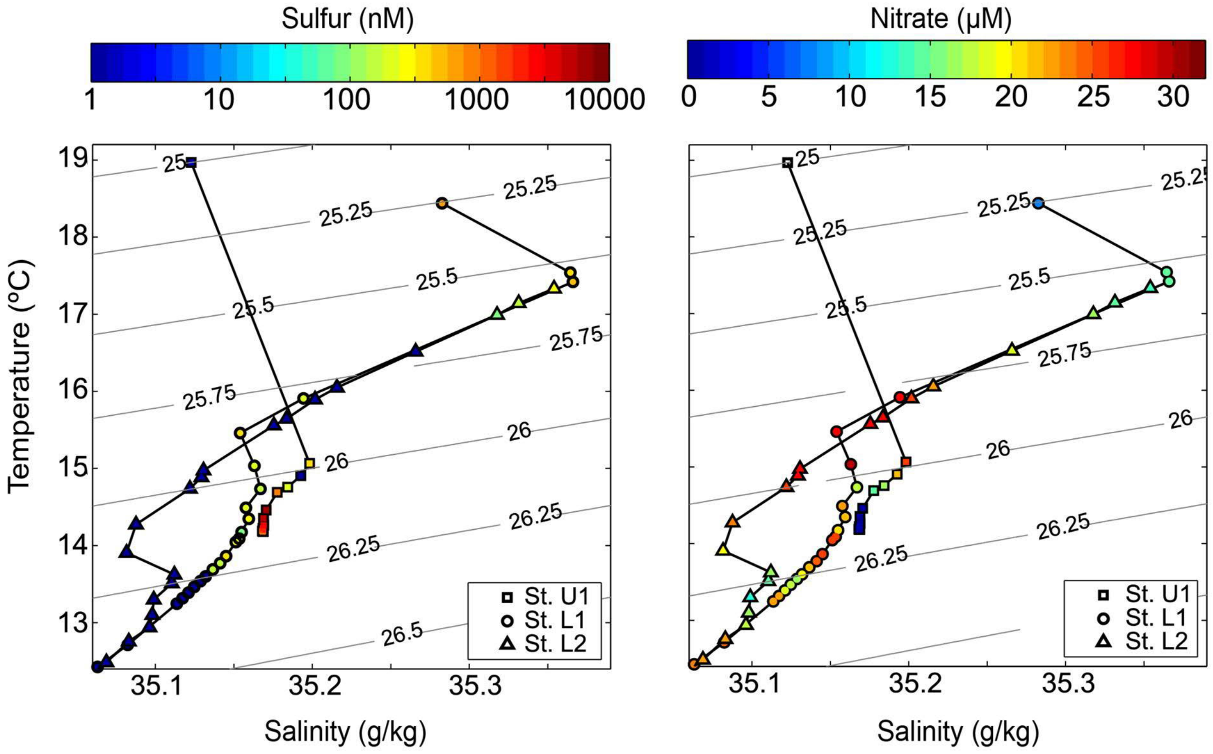

Supplementary Figure 2: Elemental sulfur and nitrate concentrations as a function of temperature-salinity for Stations U1, L1 and L2. Densities (kg m^{-3}) are indicated by the light gray isopycnals.

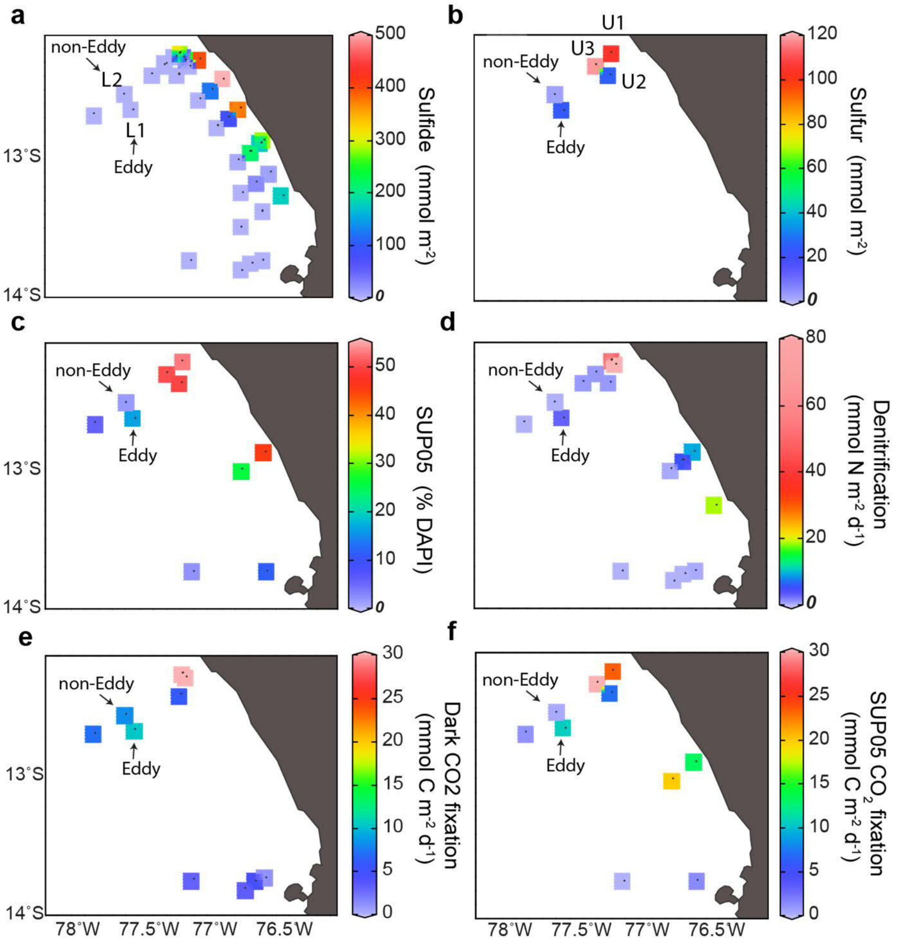

Supplementary Figure 3. Distribution of inventories and rates of (a) dissolved sulfide, (b) elemental sulfur, (c) % DAPI as SUP05, (d) denitrification, (e) dark CO_2 fixation, and (f) SUP05 CO_2 fixation. Depths of integration are from 10 m down to the sediments for coastal stations from 10 m down to 300 m depth for offshore stations, with the exception of the dark CO_2 fixation rates that are integrated from 30-70 m for coastal stations and from 100-300 m for offshore stations. In panel c the highest SUP05 abundance is reported for the respective stations.

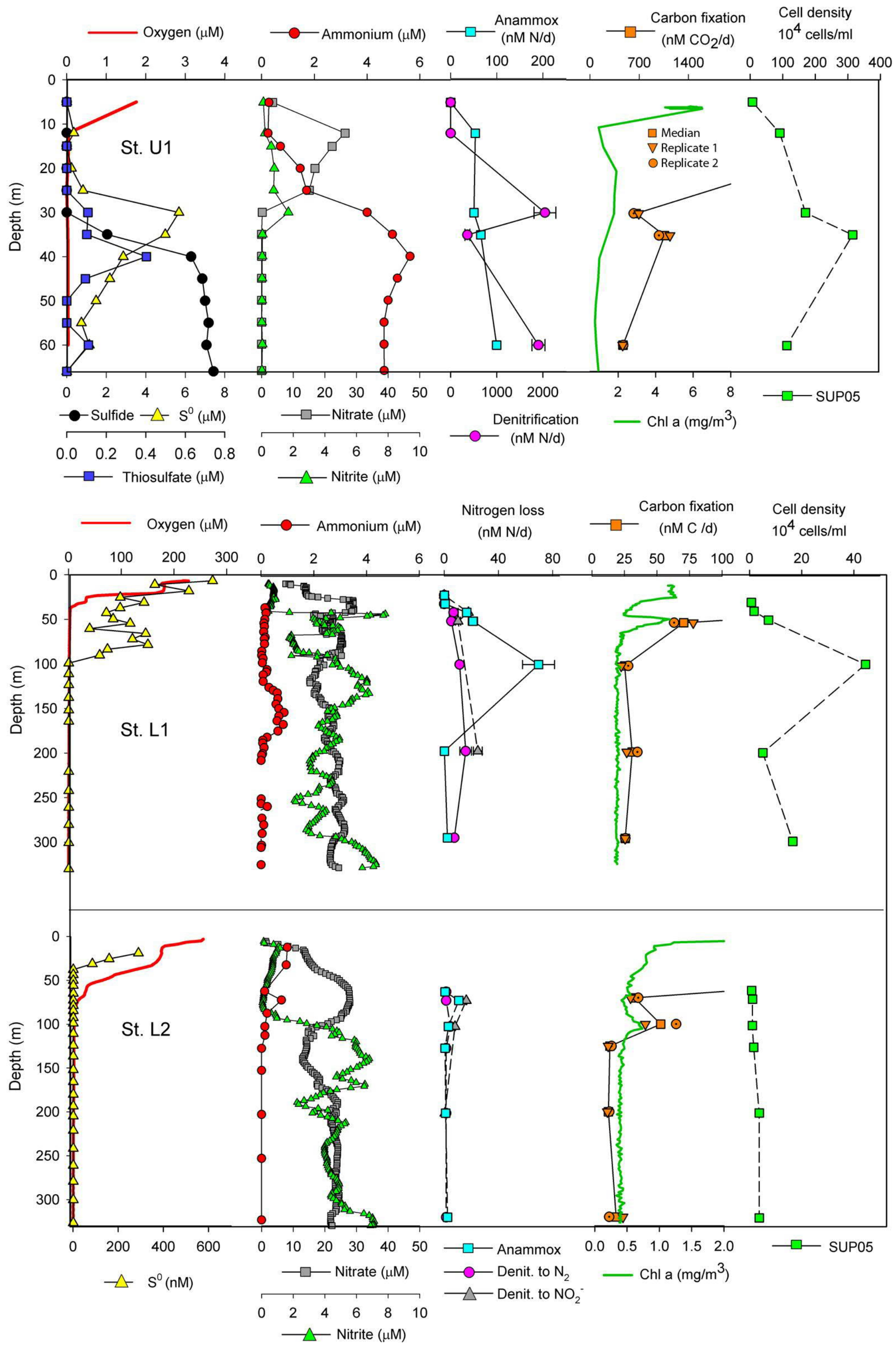

Supplementary Figure 4. Depth distributions of dissolved oxygen, key sulfur and nitrogen species, chlorophyll a, SUP05 cell densities (GSO131 probe), and rates of dark carbon fixation and dissimilatory nitrogen transformations at the three main stations U1, L1, and L2. Error bars for nitrogen transformation rates represent the standard error and were estimated according to the slope of the N_2 production rate (see Material and Methods). Note that thiosulfate was not detected at stations L1 and L2 (<50 nM). Data for Station U1 after Martinez-Perez et al. [2]

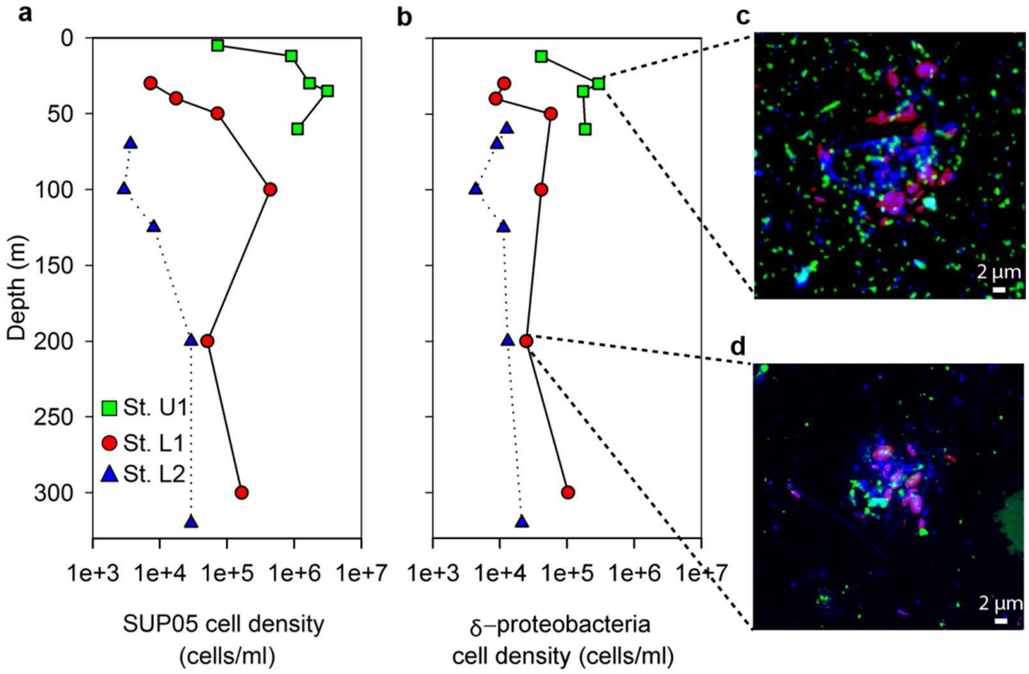

Supplementary Figure 5. CARD-FISH quantification of the distribution of SUP05 (GSO131) and deltaproteobacteria (Delta495): depth profiles of (a) SUP05 and (b) deltaprotebacteria cell densities. (c, d) CARD-FISH image of planktonic and aggregate-associated SUP05 and deltaproteobacteria from two samples. Blue-green stained cells (also marked with green arrows) represent SUP05 bacteria hybridized with the GSO131 probe. Blue shows all cells marked with the DNA stain 4′,6-diamidino-2-phenylindole (DAPI). Red stained cells represent deltaproteobacteria hybridized with the Delta495 probe [3].

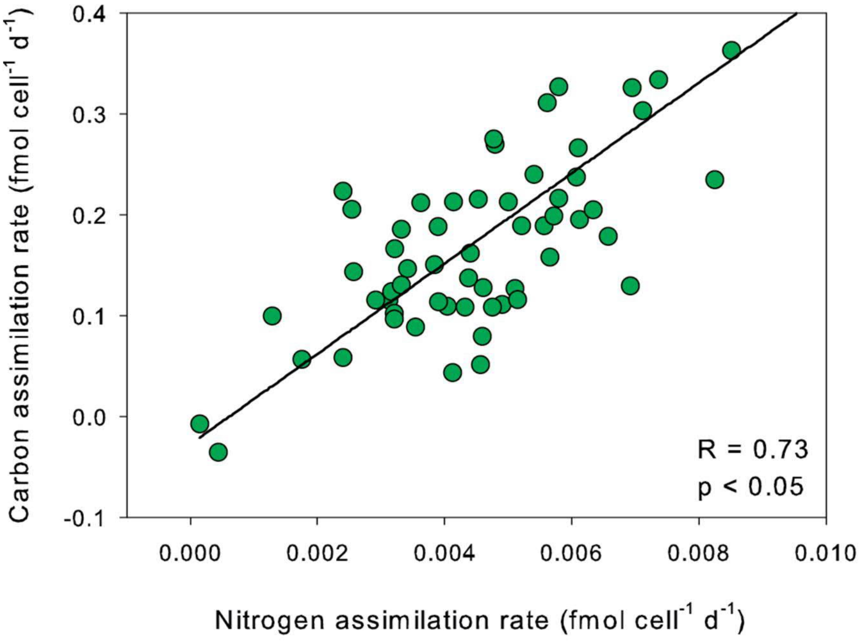

Supplementary Figure 6: Single-cell SUP05 carbon and nitrogen assimilation rates for station U1 (60 m depth).

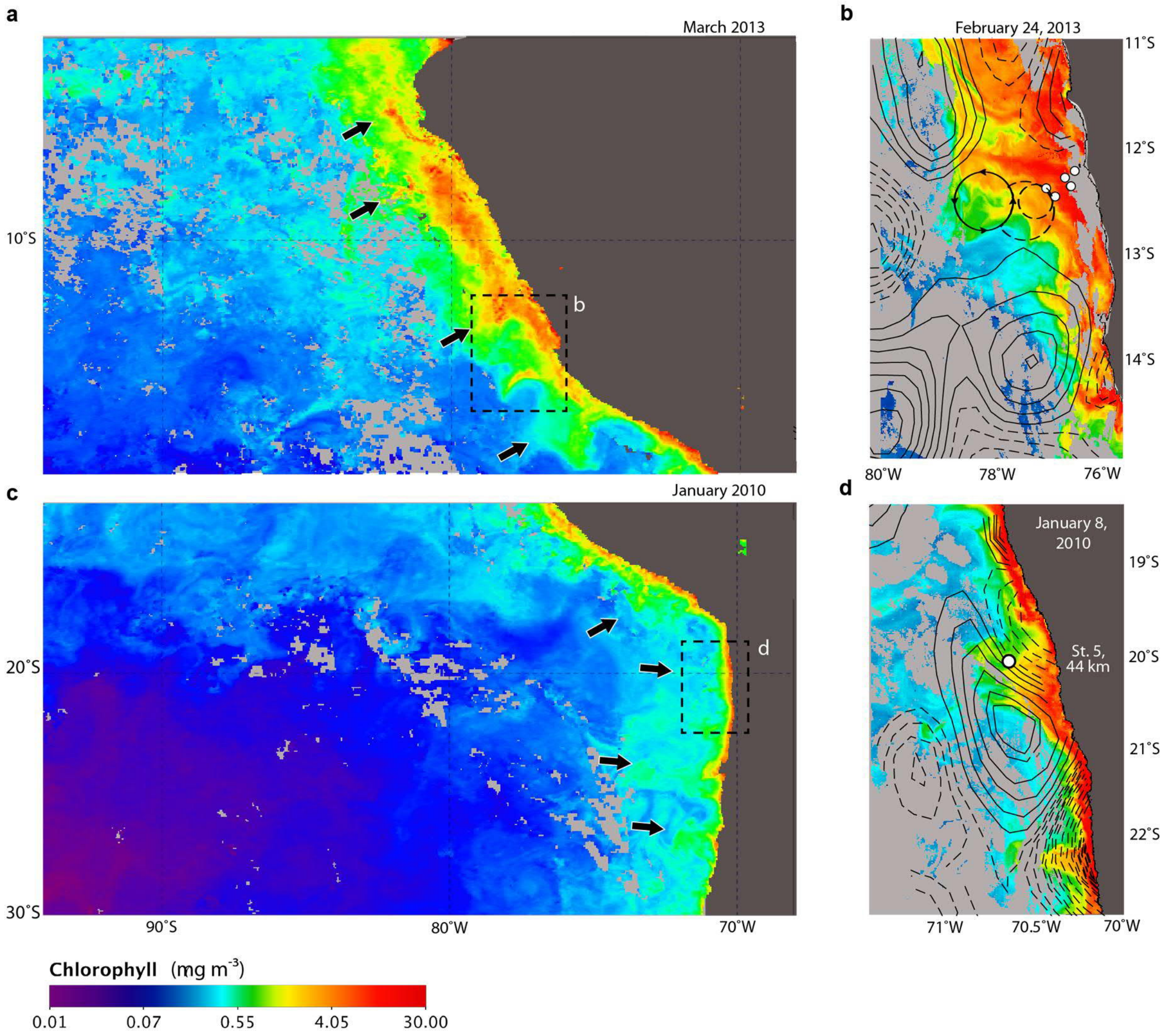

Supplementary Figure 7. Distribution of filaments in the Eastern Tropical South Pacific. (a) March, 2013 composite image of near-surface chlorophyll concentrations, where black arrows indicate filaments. (b) February 24, 2013 near-surface chlorophyll concentrations with satellite-sea surface height altimetry (SSHA) overlay. The contours of the subsurface eddy (not detectable by SSHA but detectable based on horizontal velocities is illustrated. (c) January, 2010 composite of near-surface chlorophyll concentrations (d) January 8, 2010 near-surface chlorophyll concentrations with SSHA overlay. Station 5 (white circle) located 44 km from the coast was sampled January 9th, 2010 [4].

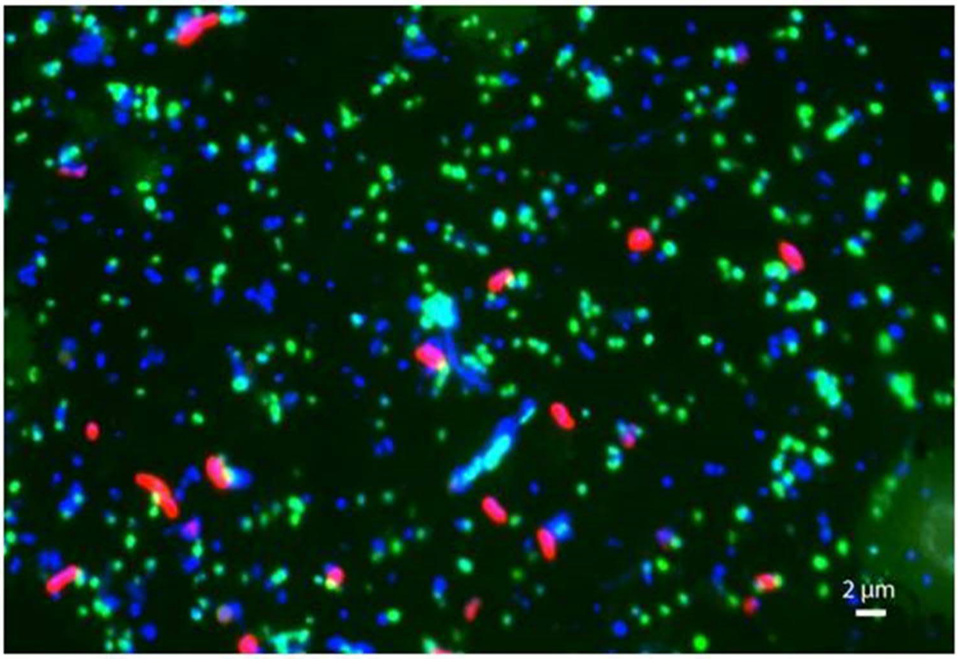

Supplementary Figure 8: GSO131 (green) and GSO1290 (red) double CARD-FISH hybridization performed at station U1 (30 m depth). Blue shows all cells marked with the DNA stain 4′,6-diamidino-2-phenylindole (DAPI).

Supplementary Table 1: List of stations sampled during the M93 research cruise February-March, 2013.

Abbreviated station name (used in text)	M93 station name	Date and time sampled	Latitude (°N)	Longitude (°E)
U2	295	Feb 9, 02:02	-12.38	-77.19
L1	318	Feb 11, 11:40	-12.64	-77.53
378	378	Feb 18, 17:04	-13.75	-76.64
L3	391	Feb 20, 21:04	-12.67	-77.82
L2	399	Feb 22, 12:23	-12.52	-77.60
U3	412	Feb 24, 10:00	-12.31	-77.30
U1a	413	Feb 25, 01:00	-12.23	-77.18
U1	471	Mar 4, 09:50	-12.23	-77.18

Supplementary Table 2: Summary of PCR primers and fluorescence in situ hybridization probes used in this study.

Target group	Primer/ probe	Sequence (5' to 3')	Size(bp)	Annealing temp/ formamide conc.	Ref.
Catalysed reported deposition–fluorescence in situ hybridization probes[1]					
SUP05	GSO131[2]	CTA TCC CCC ACT ATC TGG TAG A	22	46°C / 35% [3]	This study
Delta-proteobacteria	Del495a[4]	AGT TAG CCG GTG CTT CCT	18	46°C / 30%	[3]
	Del495b[4]	AGT TAG CCG GCG CTT CCT	18	46°C / 30%	[3]
	Del495c[4]	AAT TAG CCG GTG CTT CCT	18	46°C / 30%	[3]
Polymerse chain reaction primers					
Universal	GM3f	AGA GTT TGA TCM TGG C	16	50°C	[5]
Universal	GM4r	TAC CTT GTT ACG ACT T	16	50°C	[5]

[1] Primer and probe specificity were evaluated in silico using the SILVA SSU refnr 128 database. The probe coverage is evaluated in SupplementaryTable 3.

[2] Unlabeled competitor probes (C) are as follows: GSO131-c1: CTA TCC CCC ACT ATC AGG TAG A; GSO131-c2: CTA TCC CCC ACT ATC AGG CAG A. Competitor probe sequences were designed to exclude mismatch sequences indicated in SupplementaryTable 3.

[3] The different probes were tested under various formamide concentrations, the optimal is shown.

[4] Unlabeled competitors probes: cDel495a (AGT TAG CCG GTG CTT CTT), cDel495b (AGT TAG CCG GCG CTT C(T/G)T), and cDel495c (AAT TAG CCG GTG CTT CTT) were used according to [6,7].

Supplementary Table 3: FISH probe specificity and coverage. Probes were evaluated in silico using the SILVA SSU refnr 128 database. Eligible sequences are the total number of sequences within a given taxonomic group. The number of probe sequence matches is indicated; note that values indicated in parentheses represent the number of matches with a one-nucleotide mismatch. Competitor probes were designed towards the mismatch sequences (see Supplementary Table 2). Coverage represents the number of probe sequence matches divided by the number of eligible sequences expressed as a percentage.

Taxonomy	Coverage (%)	Eligible sequences	Number of probe sequence matches
GSO131 probe: 0 mismatches, total matches = 11 (1 mismatch, total matches = 95)			
Bacteria	0.002 (0.02)	526819	11 (95)
Proteobacteria	0.005 (0.04)	209486	10 (90)
Gammaproteobacteria	0.01 (0.09)	97852	10 (87)
Oceanospirillales	0.2 (1.14)	6164	10 (70)
SUP05 cluster	4.1 (12.24)	245	10 (30)
Outgroup hits: Arctic96BD-19 cluster	0 (15.82)	177	0 (28)
Outgroup hits: Other gammaproteobacteria	0 (0.03)	97852	0 (29)
Outgroup hits: Bacteroidetes	0.002 (0.006)	50630	1 (3)
Outgroup hits: Other			0 (8)
Del495a probe: 0 mismatches, total matches = 11609 (1 mismatch, total matches = 116906)			
Bacteria	2.2 (21.8)	537344	11609 (116906)
Proteobacteria	4.3 (36.5)	214092	9279 (78225)
Deltaproteobacteria	62.5 (88.9)	14649	9149 (13161)
Outgroup hits: SUP05 cluster	0.82 (90.2)	245	2 (221)
Outgroup hits: Non deltaproteobacteria			103745
Del495b probe: 0 mismatches, total matches = 1018 (1 mismatch, total matches = 51283)			
Bacteria	0.2 (9.5)	537344	1018 (51283)
Proteobacteria	0.2 (4.7)	214092	489 (10108)
Deltaproteobacteria	3.3 (66.7)	14649	484 (9765)
Outgroup hits: SUP05 cluster	0 (1.6)	245	0 (4)
Outgroup hits: Non deltaproteobacteria			534 (41518)
Del495c probe: 0 mismatches, total matches = 121 (1 mismatch, total matches = 13111)			
Bacteria	0.02 (2.4)	537344	121 (13111)
Proteobacteria	0.04 (4.8)	214092	86 (10246)
Deltaproteobacteria	0.6 (63.9)	14649	84 (9361)
Outgroup hits: SUP05 cluster	0 (1.6)	245	0 (4)
Outgroup hits: Non deltaproteobacteria			37 (3750)

Supplementary Table 4: Key enzymes identified in the SUP05-ETSP metagenome. The recovered SUP05 genome can be found under the NCBI accession number PNQY00000000.

Gene name	Function/protein
Sulfur metabolism	
soxXYZAB	Oxidation of reduced sulfur compounds
soxX	Sulfur oxidation protein
soxY	Sulfur oxidation protein
soxZ	Sulfur oxidation protein
soxA	Diheme cytochrome
soxB	Sulfate thiol esterase
soxZ	Sulfur oxidation protein
dsrA	Dissimilatory sulfite reductase
dsrB	Dissimilatory sulfite reductase
dsrEFH	Dissimilatory sulfite reductase
dsrMKJOP	Dissimilatory sulfite reductase
aprA	Adenylylsulfate reductase
aprB	Adenylylsulfate reductase
sat	Sulfate adenylyltransferase
fccA	Sulfide-binding, flavoprotein
fccB	Sulfide-binding, flavoprotein
Nitrogen metabolism	
narG	Nitrate reductase
nirS	Nitrite reductase
norB	Nitric oxide reductase
nosZ	Nitrous oxide reductase

References

1 Thomsen, S. et al. The formation of a subsurface anticyclonic eddy in the Peru-Chile Undercurrent and its impact on the near-coastal salinity, oxygen, and nutrient distributions. Journal of Geophysical Research: Oceans, 476-501 (2016).

2 Martínez-Pérez, C. et al. Metabolic versatility of a novel N2-fixing Alphaproteobacterium isolated from a marine oxygen minimum zone. Environmental Microbiology, 20, 755-768 (2017).

3 Loy, A. et al. Oligonucleotide Microarray for 16S rRNA Gene-Based Detection of All Recognized Lineages of Sulfate-Reducing Prokaryotes in the Environment. Applied and environmental microbiology 68, 5064-5081 (2002).

4 Canfield, D. E. et al. A cryptic sulfur cycle in oxygen-minimum-zone waters off the Chilean coast. Science 330, 1375-1378 (2010).

5 Muyzer, G., Teske, A., Wirsen, C. O. & Jannasch, H. W. Phylogenetic relationships of Thiomicrospira species and their identification in deep-sea hydrothermal vent samples by denaturing gradient gel electrophoresis of 16S rDNA fragments. Arch Microbiol 164, 165-172 (1995).

6 Macalady, J. L. et al. Dominant Microbial Populations in Limestone-Corroding Stream Biofilms, Frasassi Cave System, Italy. Applied and environmental microbiology 72, 5596-5609 (2006).

7 Lücker, S. et al. Improved 16S rRNA-targeted probe set for analysis of sulfate-reducing bacteria by fluorescence in situ hybridization. Journal of microbiological methods 69, 523-528 (2007).
